# Retraction Note: The Effect of Preterm Birth on Maximal Aerobic Exercise Capacity and Lung Function in Healthy Adults: A Systematic Review and Meta-analysis

**DOI:** 10.1007/s40279-023-01935-9

**Published:** 2023-09-09

**Authors:** Thomas Gostelow, Eric J. Stöhr

**Affiliations:** 1 School of Sport and Health Sciences, Cardif Metropolitan University, Cardif, UK; 2 Division of Cardiology, Department of Medicine, Columbia University Irving Medical Center, New York, NY USA; 3 COR-HELIX (CardiOvascular Regulation and Exercise Laboratory-Integration and Xploration), Institute of Sport Science, Leibniz University Hannover, Am Moritzwinkel 6, Building 1806, 30167 Hannover, Germany

**Retraction Note: Sports Medicine (2022) 52:2627–2635** 10.1007/s40279-022-01710-2

The authors have retracted this article because, after publication, concerns were raised about the appropriateness of the studies included in the meta-analysis. Re-analysis by the authors found that seven of the 11 studies included in the meta-analysis did not fully meet the criteria for inclusion or exclusion. Both authors agree with this retraction.

